# An evaluation experiment on the causal effects of differentiated medical insurance reimbursement on medical choice and medical expenses

**DOI:** 10.3389/fpubh.2026.1907092

**Published:** 2026-10-06

**Authors:** Zihan Zhao

**Affiliations:** Affiliated Stomatological Hospital of Nanchang University, Nanchang, Jiangxi, China

**Keywords:** difference-in-differences, differentiated health-insurance reimbursement, health-care provider choice, hierarchical medical system, medical expenditure

## Abstract

**Introduction:**

Uneven distribution of health-care resources and rising medical spending have kept medical-insurance reform high on China’s policy agenda. This study examines whether differentiated reimbursement can redirect patients toward lower-level providers and moderate the cost of care.

**Methods:**

We used provincial health-insurance claims, institutional operating records, and a patient-behavior survey covering December 2022 to August 2024. A difference-indifferences model was used to estimate policy effects, followed by mechanism and heterogeneity analyses.

**Results:**

Higher reimbursement rates for primary and secondary institutions increased the probability of choosing these providers. The association operated mainly through a lower out-of-pocket share and improved primary-care service capacity. Responses were stronger among patients with chronic conditions, older patients, and lower-income groups, whereas acute and severe cases continued to rely largely on tertiary hospitals. The policy also reduced average per-visit costs and individual payment burdens, although provider responses at some tertiary hospitals may attenuate the cost-control effect.

**Discussion:**

Differentiated reimbursement appears useful for supporting hierarchical care, but it is unlikely to establish a mature hierarchical medical system by itself. It should be combined with primary-care capacity building, clearer referral pathways, and payment-system reform.

## 1 Introduction

China’s health system continues to face a persistent mismatch between the distribution of medical resources and the pattern of patient demand. Many patients, including those with common or manageable conditions, prefer to seek care directly from tertiary hospitals. This behavior overcrowds high-level hospitals, leaves primary institutions underused, and weakens the practical operation of hierarchical diagnosis and treatment (1). To address this imbalance, health-insurance authorities have experimented with reimbursement schedules that vary by provider tier. By making primary and secondary care financially more attractive than tertiary care, these policies are expected to reshape patients’ choices and reduce unnecessary spending (2, 3). The underlying mechanism is straightforward: when the patient’s own payment is lower at a primary-care facility, the relative price of using that facility falls. Yet provider choice is not determined by price alone. Disease severity, household income, confidence in local doctors, access to information, transport conditions and the convenience of referral all influence where patients eventually go (4–6). Digital health-information systems and intelligent medical platforms may further influence provider choice by improving the processing, integration and interpretation of complex patient and service information (7). A credible evaluation therefore needs to move beyond the reimbursement rate itself and examine the behavioral and institutional conditions under which the policy works.

The literature on medical-insurance incentives and hierarchical diagnosis has shown that reimbursement differences can encourage a degree of patient diversion, but the size of this effect depends on context (8). Patients with chronic illnesses, common diseases or follow-up needs usually have repeated contact with the health system and are more sensitive to small changes in out-of-pocket payment. Their care is also more suitable for primary institutions. By contrast, patients facing acute or high-risk conditions place greater weight on specialist expertise, equipment, perceived safety and the reputation of physicians, so a higher reimbursement rate at the primary level may not be sufficient to alter their choice (9–11). Provider behavior also matters. If tertiary hospitals lose part of their patient volume, they may respond by increasing the use of diagnostic tests, procedures or high-value services in order to stabilize revenue. Such supply-side responses can dilute the intended savings of insurance reform. For this reason, the present study treats differentiated reimbursement as a joint demand-side and supply-side intervention rather than a narrow price adjustment.

Against this background, this paper evaluates a provincial differentiated-reimbursement policy implemented in China. Due to data confidentiality requirements, the province name and specific policy document are anonymized, while the implementation period and reimbursement adjustment framework are retained to clarify the institutional context. The analysis combines health-insurance settlement records, operating data from medical institutions and a survey of patients’ health-care behavior. The empirical logic is organized around four questions: whether the policy changes patients’ provider choice; whether it reduces spending and individual payment burdens; through which mechanisms the effect is transmitted; and for which groups the policy is more or less effective. A difference-in-differences design is used to estimate the net policy effect on primary-care choice and expenditure outcomes. Heterogeneity is then examined by disease type, income level and urban–rural status. Finally, mediation analysis is used to assess the roles of out-of-pocket burden and primary-service supply, while the possible adjustment of tertiary hospitals is discussed as a constraint on long-term cost control. By combining demand-side incentives with supply-side capacity in one framework, the study applies a quasi-experimental design with mechanism and heterogeneity analyses to provide empirical evidence for improving reimbursement design, strengthening hierarchical care and using medical resources more efficiently.

Figure 1 positions this study relative to the existing literature. Rather than focusing only on demand-side incentives, the analysis also considers service capacity, group and regional variation, and expenditure outcomes.

**Figure 1:**
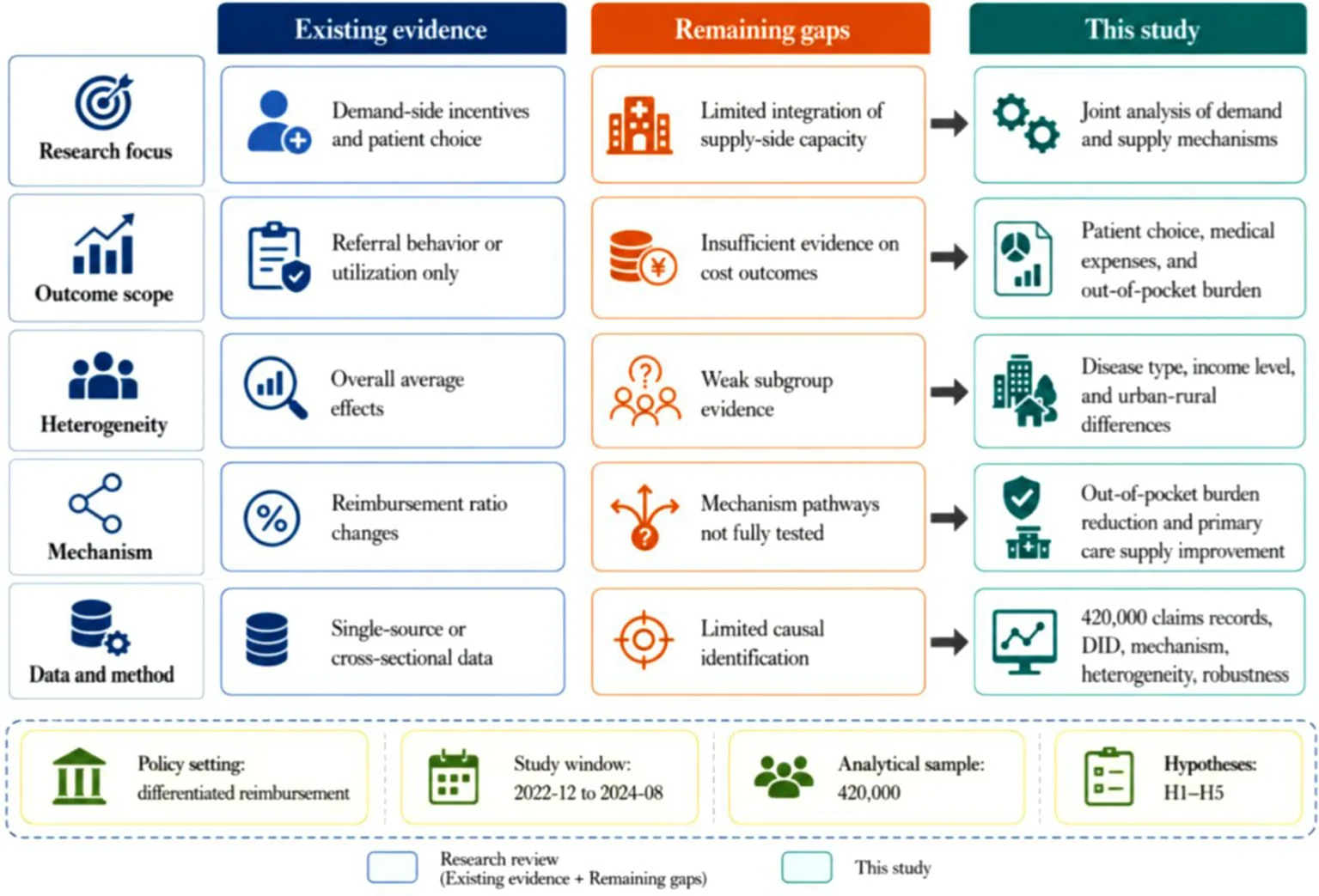
Literature gap and contribution of the study.

## 2 Literature review

### 2.1 Differentiated reimbursement and patients’ provider choice

Changing the reimbursement rate is a direct economic instrument through which medical insurance can influence provider choice. In the standard demand framework, patients compare the expected quality and convenience of different institutions with the price they personally face. When reimbursement is more generous at primary or secondary facilities, the patient’s effective price of seeking lower-level care falls, and the probability of choosing these providers should increase (12–14). However, recent evidence from China’s payment and hierarchical-care reforms shows that insurance incentives rarely operate in a mechanical or linear way. The effect is shaped by the availability of primary-care resources, patients’ disease profiles and trust in the quality of local services (15, 16).

From the patient perspective, the most responsive groups are likely to be those with chronic diseases, common conditions and planned follow-up visits. These patients use care more frequently, face cumulative payment burdens and often require continuous management rather than advanced tertiary technology (17). For severe or emergency cases, the decision calculus is different. Patients and families tend to prioritize diagnostic accuracy, equipment, specialist experience and perceived safety. Thus, even when the reimbursement advantage of primary care is increased, they may continue to choose tertiary hospitals. The effectiveness of differentiated reimbursement therefore depends not only on the financial gap between institutions, but also on whether patients believe that lower-level providers can deliver stable, safe and continuous care.

Beyond economic incentives and disease characteristics, healthcare decisions may also be influenced by physical and psychological vulnerability. Recent evidence suggests that frailty and depressive symptoms jointly contribute to health risks among aging populations, while socioeconomic disadvantage is associated with depressive symptoms and health-related behaviors (18, 19). These findings suggest that differences in health vulnerability and social conditions should also be considered when interpreting heterogeneous responses to reimbursement incentives.

### 2.2 Primary-care capacity and hierarchical diagnosis

A functioning hierarchical medical system requires demand-side incentives to be matched by supply-side capability. If primary institutions lack qualified doctors, essential equipment, medicines or chronic-disease management capacity, patients may bypass them despite lower out-of-pocket costs (20–22). Studies of China’s primary-care reform show that primary-service construction can improve access and utilization, but resource endowments and service capacity still vary substantially across regions (23). Differentiated reimbursement should therefore be analysed within a broader system that includes primary-care investment, medical alliances and referral rules, rather than treated as an isolated price lever. Previous studies further suggest that financial incentives alone are insufficient for achieving sustainable hierarchical healthcare reform. Patient trust in primary-care providers, perceived service quality and accessibility are essential conditions for maintaining patient participation in lower-level care. Strengthening primary-care capacity therefore requires not only reimbursement adjustment but also improvements in service quality, continuity of care and referral coordination (24, 25).

Urban–rural differences further complicate the policy effect. Urban residents generally have more institutional choices, better information channels and easier access to nearby primary providers. Rural residents may be more price-sensitive, but they also face longer travel distances, weaker information transmission and, in some places, thinner primary-care capacity (26, 27). The same change in reimbursement can therefore generate different behavioral responses across locations. Where local primary resources remain weak, the reimbursement incentive may not translate into actual changes in patient flow.

### 2.3 Medical expenditure and provider-side responses

Cost containment is another important objective of differentiated reimbursement. If patients with suitable conditions shift from tertiary hospitals to primary or secondary facilities, average per-visit costs should fall, and both patient payment and insurance-fund expenditure may decline. Nevertheless, expenditure is influenced not only by where patients go but also by how providers respond. The theory of supplier-induced demand suggests that, when revenue is constrained or payment rules change, providers may adjust service intensity, expand diagnostic testing or increase the use of higher-value items (28–30). Evidence from China’s hospital and payment reforms similarly indicates that hospitals may alter case mix, treatment intensity or service portfolios in response to regulation.

This means that differentiated reimbursement may generate two opposing effects. Patient diversion toward lower-level institutions can reduce average spending, while strategic behavior by high-level hospitals may offset part of that reduction. Without supporting reforms such as DRG/DIP payment, total budget control, clinical pathway management and quality monitoring, reimbursement gaps alone may have limited ability to restrain long-term expenditure growth (31–33). The present study therefore examines provider choice, expenditure changes and the possible response of tertiary hospitals within the same analytical frame.

### 2.4 Summary of the literature

Existing research offers a useful foundation for understanding differentiated reimbursement, but several gaps remain. First, many studies emphasize patient price incentives but pay less attention to primary-service capacity and provider-side adjustment. Second, average policy effects are often reported without sufficient analysis of heterogeneity by disease type, income level or urban–rural status. Third, the mechanisms linking reimbursement to provider choice and expenditure, especially through out-of-pocket burden and service supply, are still not fully explained. This paper addresses these gaps by building a framework that connects demand-side price incentives, supply-side capacity and expenditure outcomes, and by identifying policy effects through a difference-in-differences design and mediation analysis.

## 3 Research design

### 3.1 Data sources and sample construction

The empirical analysis uses three types of data from a Chinese province between December 2022 and August 2024: health-insurance settlement records, operating information from medical institutions and a patient-behavior survey. The province and policy name are anonymized due to data confidentiality requirements imposed by the data provider and ethical approval conditions. The policy was implemented in January 2024, and the study retains the implementation period, reimbursement adjustment framework and coverage criteria to ensure analytical transparency. The claims data provide the core basis for causal identification and include visit date, institution tier, diagnostic code, total expenditure, insurance payment and individual payment. These variables allow the study to observe patient flows and payment burdens before and after the policy. The institutional data include provider tier, number of beds, licensed doctors, monthly service volume and indicators of primary-care capacity. The survey, based on stratified random sampling and 800 valid responses, supplements the administrative data with information on policy awareness, care preferences, referral experience and perceived quality of primary services (Figure 2).

**Figure 2:**
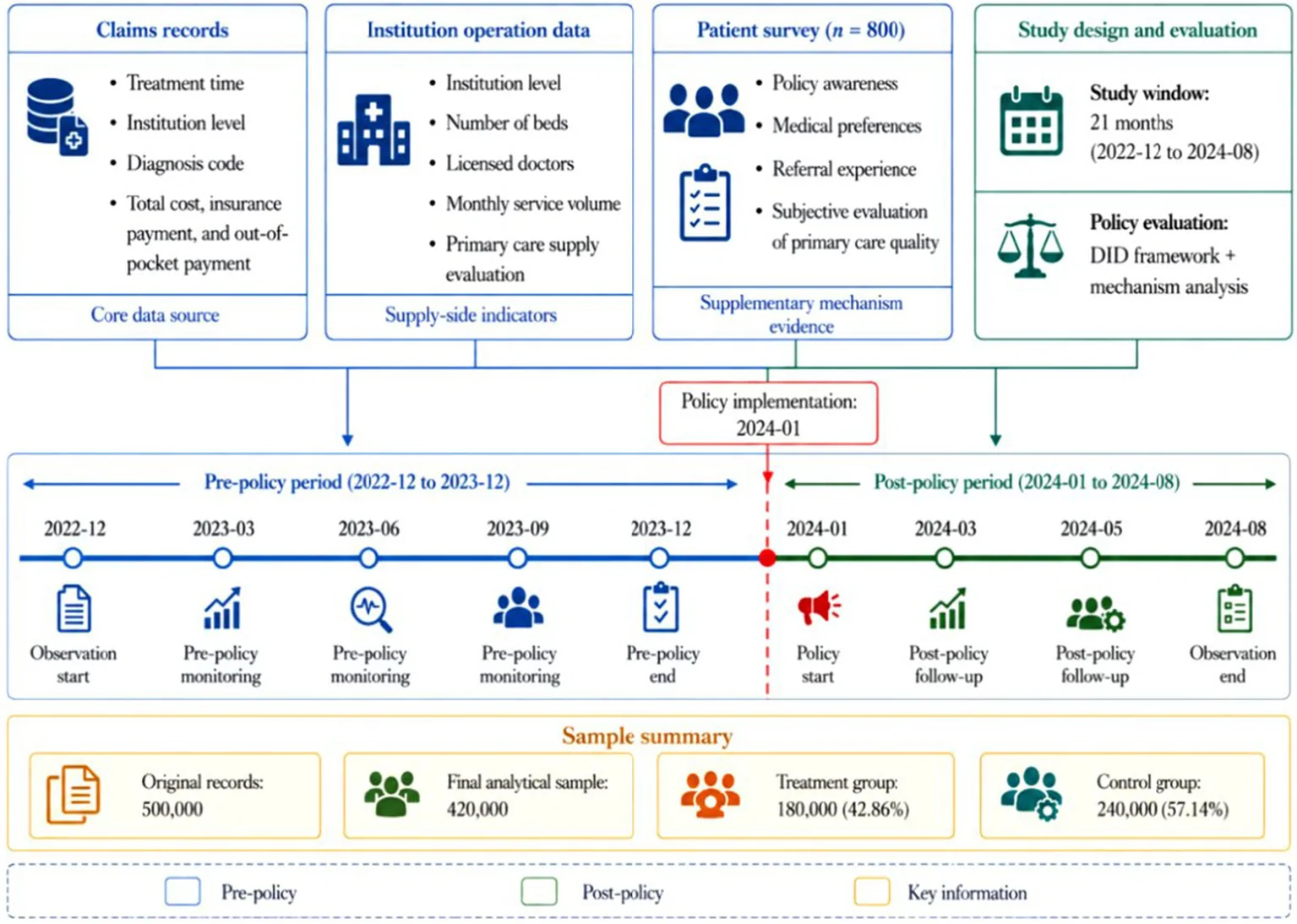
Policy timeline and study window.

Sample construction proceeded in several steps. Records with missing information on institution tier, visit time, diagnostic code or expenditure were first removed. Observations with clear logical errors, such as non-positive total costs, individual payment greater than total cost or negative out-of-pocket amounts, were then excluded. Disease categories were identified using ICD-10 codes and grouped into chronic disease, acute disease and severe acute disease. The baseline regressions rely mainly on the claims data, while the patient survey is used for mechanism interpretation and supplementary analysis. This design avoids letting the smaller survey sample drive the central causal estimates. After cleaning and matching, the final dataset is organized at the patient-visit-institution-time level and used for DID estimation, heterogeneity tests and robustness checks. Multiple visits from the same patient are retained as separate observations, and individual fixed effects are included to control for time-invariant patient characteristics.

As shown in Figure 3, the original claims records were screened for missing values and cost anomalies, and the resulting 21-month sample was then split into treatment and control groups.

**Figure 3:**
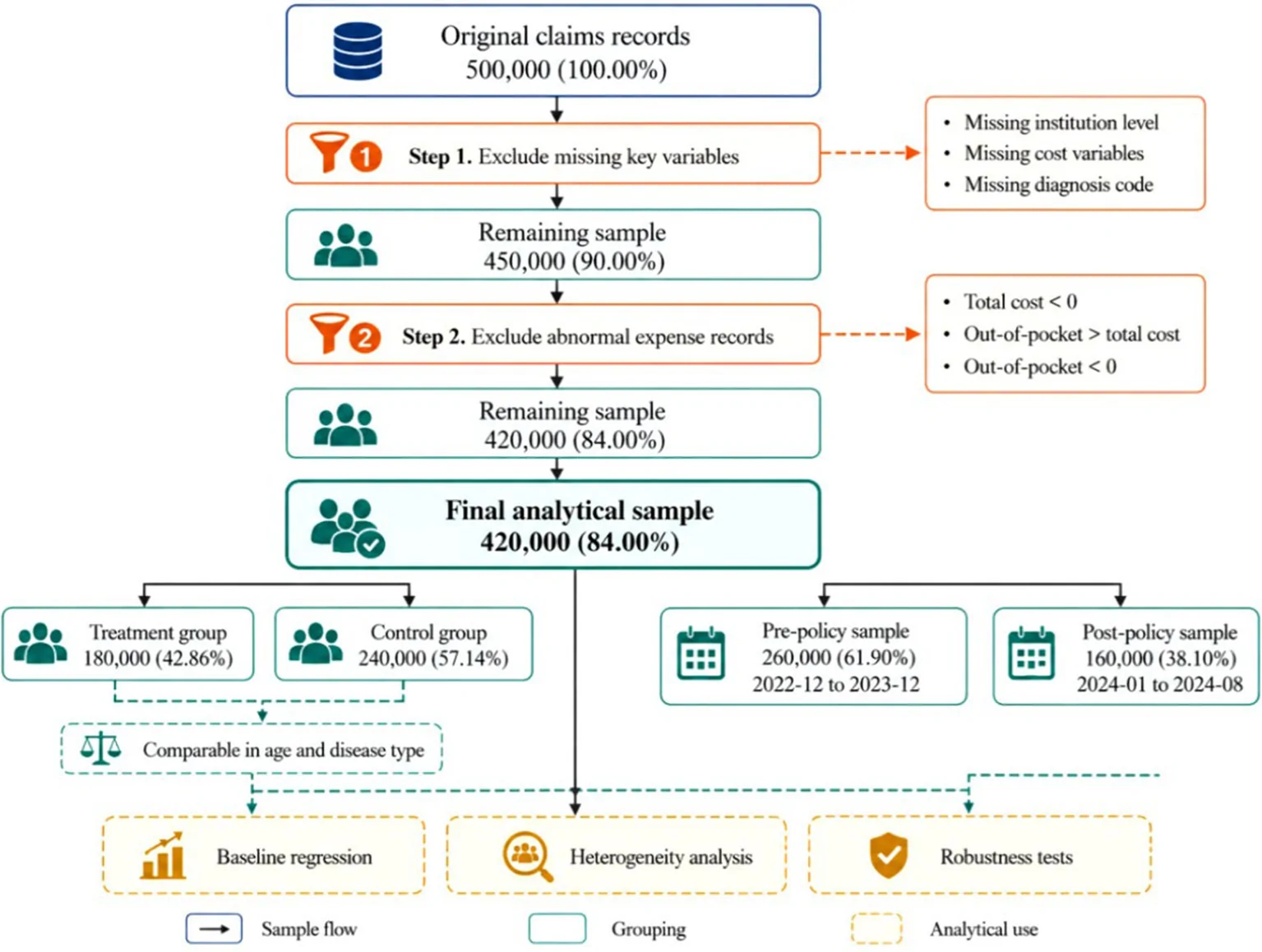
Sample construction process.

### 3.2 Variable definition

The key policy variable is the differentiated reimbursement reform. The post-policy indicator, post, equals one after policy implementation and zero before implementation. The treatment status is assigned at the regional policy level according to the geographical coverage of the differentiated reimbursement reform. The claims observations from insured individuals within covered regions are classified as the treatment group, while individual treatment assignment is not assumed. The Treat×Post interaction captures the differential change after policy implementation. The interaction term $T\mathrm{rea}t_{i}\times \mathrm{Pos}t_{t}$ captures the differential change after implementation and identifies the policy effect on provider choice and expenditure outcomes.

The dependent variables cover both medical choice and expenditure. Provider choice is measured by PrimaryChoice_it, which equals one when a visit occurs at a primary or secondary institution and zero when it occurs at a tertiary hospital. Expenditure outcomes include total per-visit cost, insurance-fund payment, individual out-of-pocket payment and the out-of-pocket ratio. The out-of-pocket ratio is calculated as individual payment divided by total medical expenditure. Because medical costs are right-skewed, total per-visit cost is also transformed using the natural logarithm.

Control variables include patient characteristics, disease attributes and institutional features. Patient-level controls consist of gender, age and insurance status. Disease controls distinguish chronic disease, acute disease and severe disease. Institutional controls include provider tier, beds, licensed doctors and monthly service volume. Income groups were classified according to annual household income categories reported in the patient survey, while urban–rural status was identified based on patients’ registered residence information. These classifications were used only for heterogeneity analysis. These variables are included for heterogeneity analysis. Two mechanism variables are used: the patient’s out-of-pocket ratio and the primary-service supply index. The former tests the economic-burden channel, while the latter tests whether improved primary-care capacity strengthens patient diversion.

Figure 4 links the outcome variables, treatment indicators and mechanism variables to the descriptive analysis, DID estimation, mechanism test, heterogeneity analysis and robustness checks.

**Figure 4:**
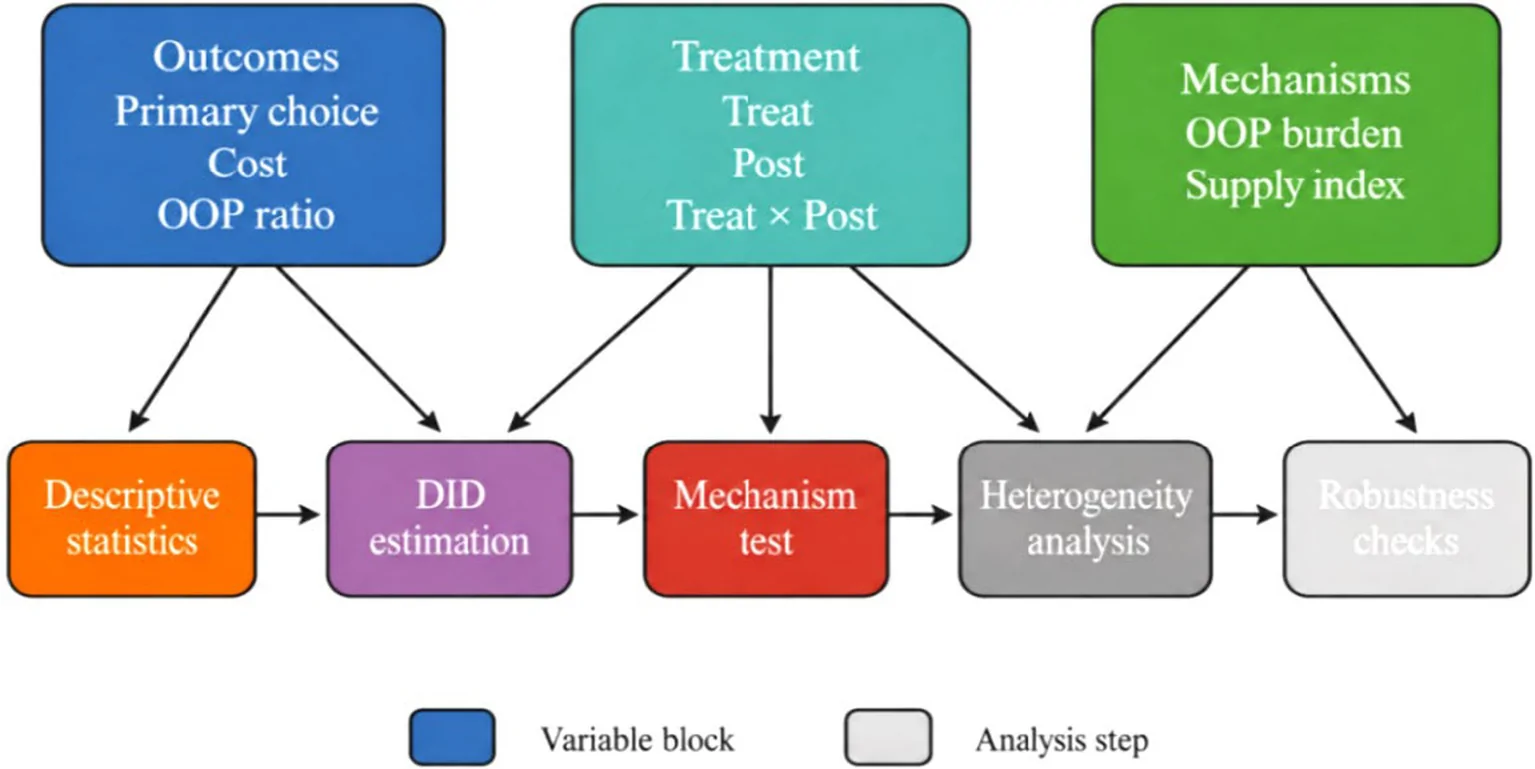
Variable structure and empirical strategy.

### 3.3 Research hypotheses

The empirical analysis tests the following five hypotheses, which are summarized in Figure 5:

**Figure 5:**
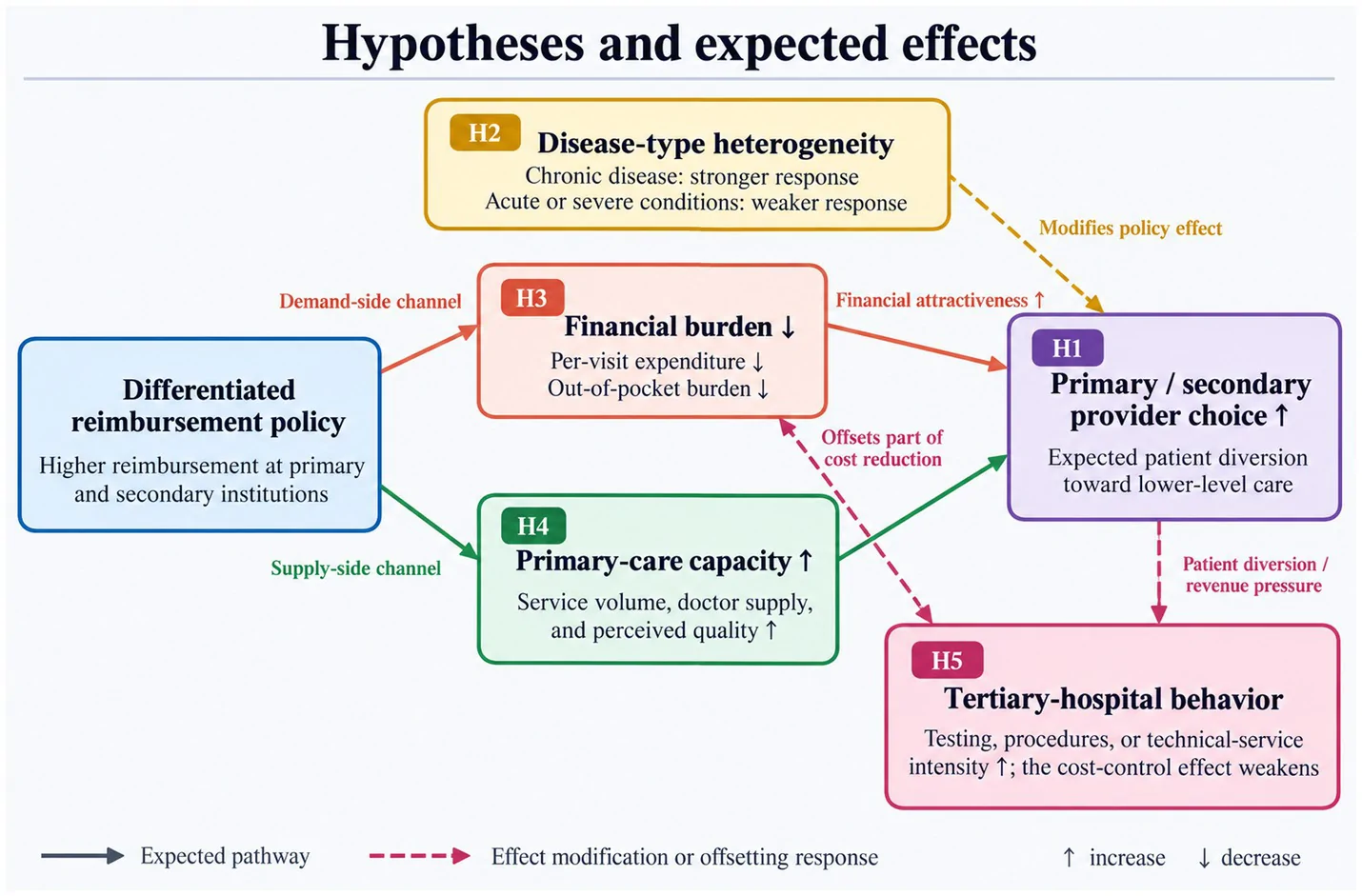
Hypotheses and expected effects.

*H1*: Differentiated health-insurance reimbursement increases the likelihood that patients choose primary or secondary medical institutions. A higher reimbursement rate at lower-level providers reduces the patient’s payment burden and increases the financial attractiveness of primary care (34).

*H2*: The policy effect is stronger for patients with chronic diseases than for patients with acute or severe conditions. Chronic-disease care involves repeated visits and long-term management, making patients more sensitive to price changes and more suitable for primary-care management, whereas severe cases depend more heavily on tertiary technology and equipment (35).

*H3*: Differentiated reimbursement reduces per-visit expenditure and the patient’s out-of-pocket burden. When appropriate cases move to lower-level providers, the average service price and intensity of treatment are expected to decline, leading to lower total costs and a lower individual payment share (36).

*H4*: Primary-care service capacity mediates the relationship between reimbursement policy and patient choice. If service volume, doctor supply and perceived quality improve at the primary level, the reimbursement incentive should have a stronger effect; if capacity is weak, the policy impact will be limited (35).

*H5*: Behavioral responses by tertiary hospitals may weaken the cost-control effect. Facing patient diversion and revenue pressure, tertiary hospitals may increase the intensity of tests, procedures or technical services, thereby offsetting part of the expenditure reduction.

### 3.4 Empirical model

To estimate the policy effect, this paper applies a difference-in-differences specification. By comparing changes in the treatment and control groups before and after implementation, the model removes common time shocks and part of the unobserved time-invariant differences between groups. Because treatment exposure is assigned at the regional policy level, the DID identification relies on the interaction between regional treatment status and the post-policy period, while individual fixed effects control for time-invariant patient characteristics. The coefficient of Treat×Post is therefore identified from changes in outcomes across regions with different policy exposure over time rather than from within-person variation in Treat. The baseline specification is presented in Equation 1:

$$Y_{\mathrm{it}}=\alpha +\beta_{1}\text{Trea}t_{i}\times \mathrm{Pos}t_{t}+\beta_{2}X_{\mathrm{it}}+\mu_{i}+\lambda_{t}+\varepsilon_{\mathrm{it}}$$ (1)

In this equation, $Y_{\mathrm{it}}$ denotes the outcome for patient i at time t, including lower-level provider choice, per-visit cost and out-of-pocket ratio. $\text{Trea}t_{i}$ identifies the treatment group, $\mathrm{Pos}t_{t}$ identifies the post-policy period, and $\text{Trea}t_{i}\times \mathrm{Pos}t_{t}$ is the DID term of interest. The coefficient $\beta_{1}$ measures the average treatment effect of differentiated reimbursement. $X_{\mathrm{it}}$ is the vector of controls, $\mu_{i}$ represents individual fixed effects, $\lambda_{t}$ represents time fixed effects, and $\varepsilon_{\mathrm{it}}$ is the error term. A positive $\beta_{1}$ in the provider-choice model implies that the policy increases lower-level care use; a negative $\beta_{1}$ in the cost model implies a reduction in expenditure or patient burden.

The identifying assumption for DID is the parallel-trends condition. To assess it, a dynamic DID model is used to compare the pre-policy trajectories of the treatment and control groups and to observe the post-policy evolution of the effect. To assess the paralleltrends condition, we estimate the dynamic DID model shown in Equation 2:

$$Y_{\mathrm{it}}=\alpha +\sum_{k}\beta_{k}\text{Trea}t_{i}\times D_{k}^{t}+\gamma X_{\mathrm{it}}+\mu_{i}+\lambda_{t}+\varepsilon_{\mathrm{it}}$$ (2)

Here, $D_{k}^{t}$ denotes dummy variables for periods relative to policy implementation, and $\beta_{k}$ traces the dynamic effect in each period. If the pre-policy coefficients are statistically indistinguishable from zero, the treatment and control groups do not display systematic trend differences before the reform. Significant coefficients after implementation would suggest that the policy effect emerges or accumulates over time.

The mechanism analysis is conducted from both demand and supply perspectives. The demand-side channel asks whether the policy changes provider choice by lowering patients’ out-of-pocket burden. The supply-side channel asks whether better primary-service supply increases the attractiveness of lower-level institutions. The demand- and supply-side mechanism models are presented in Equation 3:

$$M_{\mathrm{it}}=\alpha +\theta_{1}\text{Trea}t_{i}\times \mathrm{Pos}t_{t}+\theta_{2}X_{\mathrm{it}}+\mu_{i}+\lambda_{t}+\varepsilon_{\mathrm{it}}$$

$$Y_{\mathrm{it}}=\alpha +\delta_{1}\text{Trea}t_{i}\times \mathrm{Pos}t_{t}+\delta_{2}M_{\mathrm{it}}+\delta_{3}X_{\mathrm{it}}+\mu_{i}+\lambda_{t}+\varepsilon_{\mathrm{it}}$$ (3)

$M_{\mathrm{it}}$ denotes the mechanism variable, either the out-of-pocket ratio or the primary-service supply index. If $\theta_{1}$ is significant, the policy affects the mechanism. If the mechanism variable is significant in the outcome equation and the coefficient of $\text{Trea}t_{i}\times \mathrm{Pos}t_{t}$ becomes smaller after the mechanism is added, the evidence supports partial mediation.

### 3.5 Heterogeneity analysis

Patients may respond differently to reimbursement incentives, so heterogeneity is examined along three dimensions: disease type, income level and urban–rural status. Firstly, the sample is divided into chronic disease, acute disease and severe acute disease. Chronic-disease patients require repeated management and are expected to be more sensitive to cost changes. Severe cases, by contrast, depend more on specialist expertise, equipment and emergency capacity, so reimbursement differences may have weaker influence on their choices. Secondly, patients are grouped by income level. Lower-income patients generally face tighter budget constraints and may respond more strongly to out-of-pocket differences, whereas higher-income patients may attach greater importance to perceived quality and provider tier. Thirdly, the sample is divided by urban and rural residence. A weaker rural response would suggest that travel distance, information barriers or limited primary-care capacity reduce the practical effect of reimbursement incentives.

This heterogeneity analysis helps move beyond the average treatment effect. It identifies the boundary conditions under which differentiated reimbursement is most effective and provides evidence for more targeted insurance design, primary-service improvement and refined hierarchical diagnosis policy.

### 3.6 Robustness checks

Several robustness checks are used to evaluate the stability of the estimates. Firstly, a parallel-trends test examines whether the main outcome variables followed similar pre-policy paths in the treatment and control groups. Secondly, a placebo test assigns a false implementation date before the actual policy and re-estimates the model. Insignificant placebo coefficients would suggest that the main results are not driven by pre-existing trends or contemporaneous shocks unrelated to the policy. Thirdly, alternative measures of the core variables are used. The provider-choice outcome is replaced by the share of primary visits among all visits, and expenditure outcomes are replaced by insurance-fund payment, individual payment and the out-of-pocket ratio. Fourth, extreme expenditure observations are winsorized to reduce the influence of outliers. Fifth, regional and institutional fixed effects are added to account for differences in medical resources and unobserved institutional characteristics.

Together, these tests assess whether the baseline findings are sensitive to model choice, variable definition, sample treatment or unobserved regional and institutional factors.

## 4 Empirical results

Using the cleaned claims data, institutional records and patient survey information for December 2022 to August 2024, this section evaluates the implementation effect of differentiated reimbursement. The analysis first describes sample construction and the distribution of key variables, then compares pre- and post-policy changes. It next estimates the net policy effect using DID and finally reports mechanism, heterogeneity and robustness results. For readability, each table is introduced before it is presented.

### 4.1 Sample composition and descriptive statistics

The credibility of empirical analysis depends on the quality and comparability of the sample. As shown in Table 1, the original claims dataset contains 500,000 records from December 2022 to August 2024. After deleting observations that lacked institutional level, cost, or diagnostic codes, there were still 450,000 records. Further screening eliminated logically inconsistent costs, including non positive total expenditures and personal payments beyond feasible limits. The final analysis sample contains 420,000 records, equivalent to 84.00% of the original dataset. The treatment group contains 180,000 claim-level observations from insured individuals in the areas covered by the policy, accounting for 42.86% of the analytical sample. The control group contains 240,000 claim-level observations from insured individuals in non-covered areas, which are comparable with the treatment group in terms of age and disease composition. From December 2022 to December 2023, there were 260,000 pre policy observations, and from January 2024 to August 2024, there were 160,000 post policy observations. This structure supports the comparability required for DID design.

**Table 1:** Sample construction and grouping.

Sample construction step	Observations	Share of original sample	Description
Original claims records	500,000	100%	21 months from December 2022 to August 2024
Eliminate the remaining samples after missing key variables	450,000	90%	Eliminate the missing records of medical institution grade, cost and diagnostic code
Remaining samples after excluding expense exceptions	420,000	84%	Eliminate records with total expense ≤ 0, out of pocket amount>total expense or<0
Final analytic sample (claim-level observations)	420,000	84%	For baseline regression
Treatment-group observations	180,000	42.86%	Claims observations from insured individuals within the policy coverage area (directly affected by differentiated reimbursement policy)
Control-group observations	240,000	57.14%	Claims observations from insured individuals in areas not covered by the policy are comparable with the treatment group in terms of age, disease type and other characteristics
Pre-policy observations	260,000	61.90%	December 2022 to December 2023
Post-policy observations	160,000	38.10%	January 2024 to August 2024

Table 2 reports the distribution of the main variables. The mean of PrimaryChoice is 0.35, meaning that roughly 35% of visits occurred at primary or secondary institutions; this leaves substantial room for further patient diversion. Mean total per-visit cost is 1,800 yuan, and the mean of log cost is 7.50. The average individual payment is 504 yuan, while the out-of-pocket ratio averages 0.28, showing that patients still shoulder a notable share of spending. The means of Treat and Post are 0.4286 and 0.3810, matching the sample shares of the treatment group and the post-policy period. The supply index averages 5.20 after standardizing and weighting monthly service volume, licensed doctors and beds at primary institutions. Overall, the variables fall within reasonable ranges and show no abnormal dispersion.

**Table 2:** Descriptive statistics of main variables.

Variable	Observations	Mean	Std. dev.	Min	Max
PrimaryChoice	420,000	0.35	0.477	0	1
TotalCost	420,000	1800	1,200	50	10,000
OOPAmount	420,000	504	350	10	3,000
OOPRatio	420,000	0.28	0.15	0.05	0.8
Treat	420,000	0.4286	0.495	0	1
Post	420,000	0.381	0.486	0	1
Supply	420,000	5.2	1.8	2	10
Age	420,000	45	15	18	85
Gender	420,000	0.52	0.5	0	1
ChronicDisease	420,000	0.3	0.46	0	1
AcuteDisease	420,000	0.5	0.5	0	1
SevereDisease	420,000	0.2	0.4	0	1

The primary-service supply index is constructed from standardized monthly service volume, licensed doctors and beds at primary institutions, with weights of 0.4, 0.3 and 0.3. A higher value indicates stronger primary-care capacity. Standardization is performed before weighting to remove differences in measurement units.

Figure 6 provides a diagnostic view of the core variables and their pairwise relationships. Cost variables are highly correlated, PrimaryChoice is negatively related to lnCost, OOPAmount and OOPRatio, and it is positively related to Supply.

**Figure 6:**
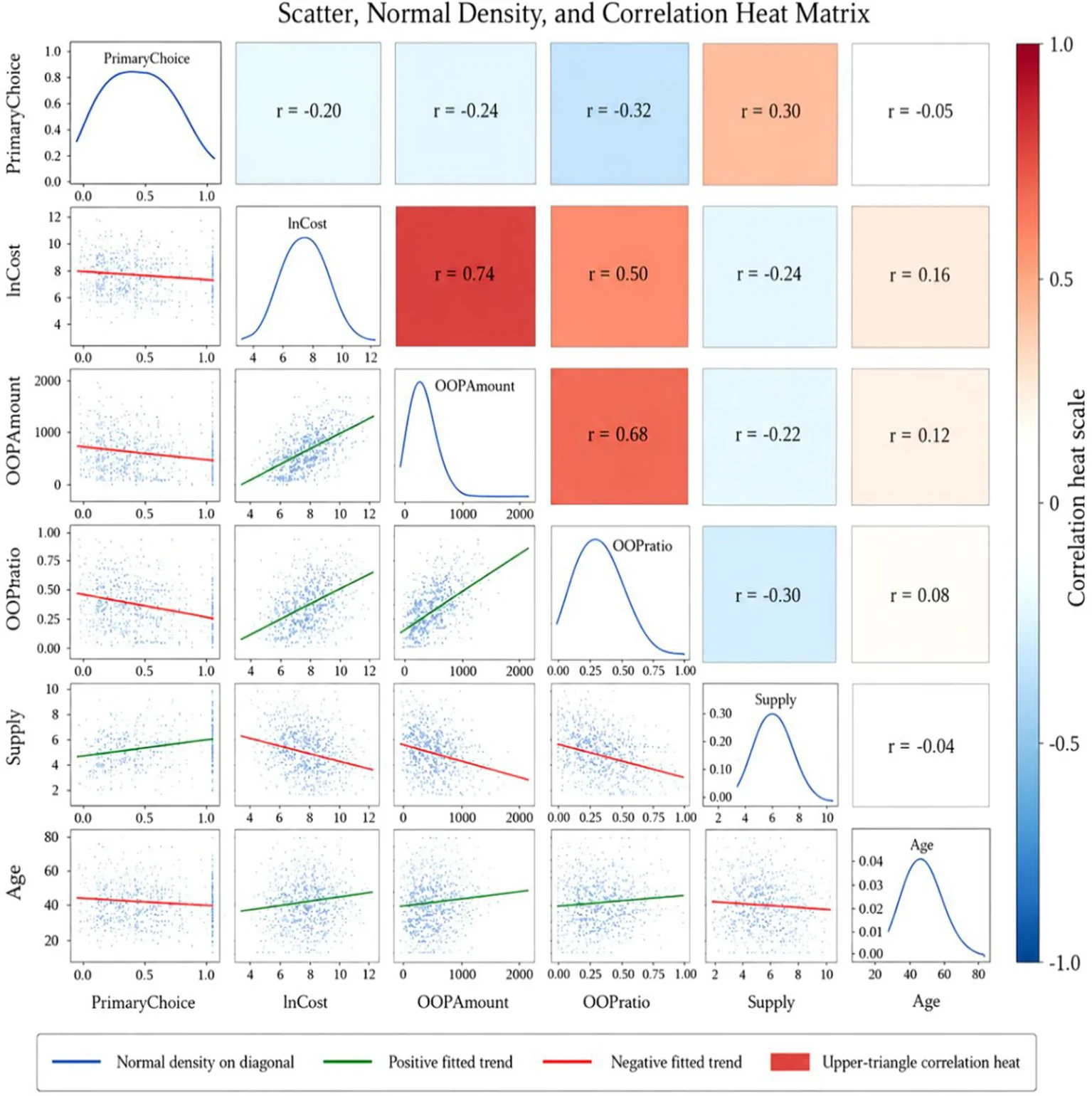
Scatter plots, density curves and correlation matrix.

Mean comparisons offer preliminary evidence on the direction of change after implementation. Table 3 shows that the share of lower-level provider choice increased from 0.32 to 0.39, a rise of 0.07 that is significant at the 1% level. During the same period, per-visit total expenditure fell from 1,900 yuan to 1,700 yuan, log cost declined by 0.10, individual payment dropped from 600 yuan to 420 yuan, and the out-of-pocket ratio decreased from 0.316 to 0.247. These changes suggest a noticeable reduction in the patient’s financial burden. The primary-service supply index increased from 5.00 to 5.50, also significant at the 1% level, which is consistent with improved local capacity. Insurance-fund payment fell slightly from 1,300 yuan to 1,280 yuan, but the difference is not significant. Thus, the policy appears to reduce patient burden without expanding fund pressure. These simple before-after comparisons are descriptive only; the DID model is needed to control for common trends and other confounders.

**Table 3:** Main variables before and after policy implementation.

Variable	Pre-policy mean	Post-policy mean	Change	t statistic	*p* value
Lower-level provider choice	0.32	0.39	0.07	12.5	<0.001
Per-visit total cost	1900	1700	−200	−8.3	<0.001
lnCost	7.55	7.45	−0.1	−9.1	<0.001
Insurance-fund payment	1,300	1,280	−20	−1.2	0.23
Individual payment	600	420	−180	−15.2	<0.001
Out-of-pocket ratio	0.316	0.247	−0.069	−18.5	<0.001
Primary-service supply	5	5.5	0.5	10.3	<0.001
Share of chronic-disease patients	0.29	0.31	0.02	3.5	<0.001
Share of acute/severe cases	0.21	0.2	−0.01	−1.8	0.07

Insurance-fund payment does not change significantly, suggesting that the reduction in patient burden is not achieved by simply increasing fund expenditure. The mean comparison is preliminary; the DID model identifies the net policy effect more rigorously.

### 4.2 Baseline regression results

Table 4 reports the effect of differentiated reimbursement on lower-level provider choice using a linear probability model. In the specification without controls, the coefficient on Treat x Post is 0.062 and significant at the 1% level. After individual characteristics, disease controls, institutional characteristics, and individual and time fixed effects are added, the coefficient declines to 0.048 but remains highly significant. This implies that, after observable and time-invariant factors are controlled for, the policy increases the probability of choosing a primary or secondary institution by about 4.8 percentage points. The control variables are consistent with expectations. Chronic-disease patients are more likely to use lower-level care, whereas acute and severe cases are less likely to do so, confirming the importance of disease severity and continuity of care in provider choice. Figure 7 illustrates the baseline DID estimates. The policy coefficient remains positive and statistically significant across specifications, while explanatory power improves after controls and fixed effects are introduced.

**Table 4:** Effect of differentiated reimbursement on lower-level provider choice (LPM).

Variable	Model 1 (uncontrolled)	Model 2 (plus individual control)	Model 3 (plus disease control)	Model 4 (plus fixed effect)
Treat×Post	0.062*** (0.008)	0.058*** (0.008)	0.055*** (0.008)	0.048*** (0.007)
Treat	−0.031** (0.012)	−0.028** (0.011)	−0.025*(0.011)	—
Post	0.045*** (0.009)	0.042*** (0.009)	0.038*** (0.009)	—
Gender	—	0.015*(0.008)	0.014* (0.008)	0.012 (0.007)
Age	—	−0.001** (0.0004)	−0.001** (0.0004)	−0.001* (0.0005)
InsuranceStatus	—	0.022*** (0.009)	0.020*** (0.009)	0.017* (0.009)
ChronicDisease	—	—	0.080*** (0.010)	0.075***(0.009)
AcuteDisease	—	—	−0.050*** (0.010)	−0.045*** (0.009)
SevereDisease	—	—	−0.120***(0.015)	−0.110*** (0.014)
Institutional controls	No	No	Yes	Yes
Individual fixed effects	No	No	No	Yes
Time fixed effects	No	No	No	Yes
Observations	420,000	420,000	420,000	420,000
R^2^	0.025	0.032	0.050	0.120

Standard errors are reported in parentheses. ***p < 0.01, **p < 0.05, *p < 0.1. In Model 4, Treat and Post are absorbed by individual and time fixed effects and are therefore not separately reported. The policy effect is identified by the Treat × Post interaction term.

**Figure 7:**
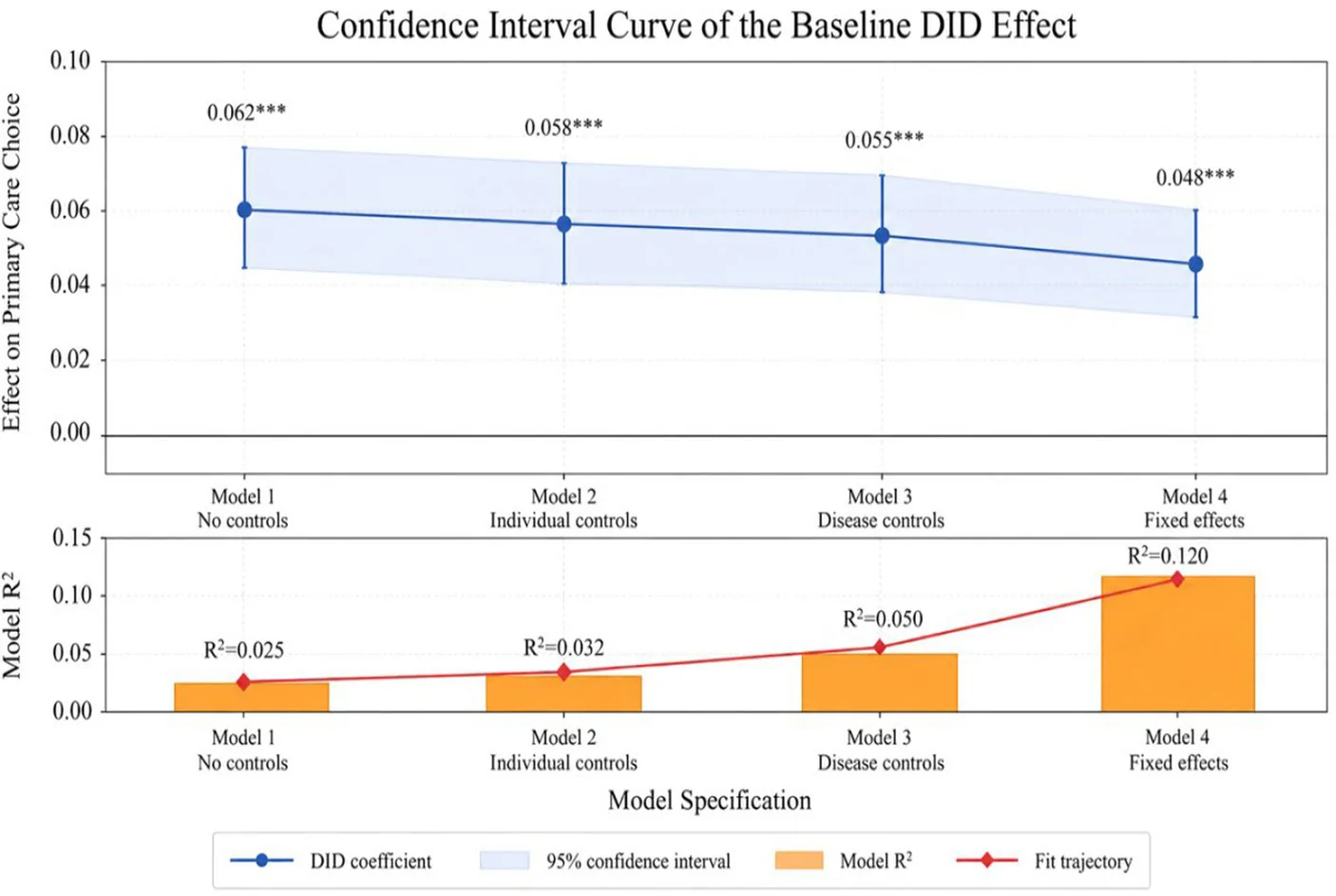
Confidence interval of the baseline DID effect.

The expenditure results are reported in Table 5. The coefficient on Treat x Post for lnCost is −0.082 and is significant at the 1% level, corresponding to an approximate 7.9% decline in per-visit expenditure. The coefficients for TotalCost and OOPAmount are −150.00 and −120.00, respectively, both significantly negative. The coefficient for OOPRatio is −0.051, meaning that the patient’s payment share falls by about 5.1 percentage points. By contrast, the coefficient for InsurancePayment is 20.00 and is not statistically significant. These findings indicate that differentiated reimbursement not only increases lower-level provider use but also improves the expenditure structure by reducing patient burden without significantly raising insurance-fund spending.

**Table 5:** Effect of differentiated reimbursement on expenditure and patient burden.

Variable	lnCost	TotalCost	OOPAmount	OOPRatio	InsurancePayment
Treat × post	−0.082*** (0.012)	−150.00*** (20.00)	−120.00*** (15.00)	−0.051*** (0.006)	20.00(18.00)
Control variables (individual + disease + institution)	Yes	Yes	Yes	Yes	Yes
Individual fixed effects	Yes	Yes	Yes	Yes	Yes
Time fixed effects	Yes	Yes	Yes	Yes	Yes
Observations	420,000	420,000	420,000	420,000	420,000
R^2^	0.150	0.140	0.160	0.180	0.130

The lnCost coefficient of −0.082 implies a reduction of approximately 7.87% because exp (−0.082)-1 is about −7.87%. Treat and Post are absorbed by fixed effects and are not displayed. The insignificant coefficient on insurance payment indicates that the main effect is cost-structure optimization and lower individual burden rather than fund expenditure expansion. *p < 0.10, **p < 0.05, and ***p < 0.01.

### 4.3 Mechanism and heterogeneity analysis

Table 6 examines the channels through which the policy affects provider choice. Model 1 shows that Treat x Post reduces OOPRatio by 0.045 at the 1% significance level, confirming that the policy lowers the patient’s payment share. Model 2 shows that the policy raises the supply index by 0.35, also significant at the 1% level, suggesting stronger primary-care capacity after implementation. When OOPRatio and Supply are added to the PrimaryChoice model, the former is significantly negative and the latter is significantly positive. The policy coefficient falls from the baseline value of 0.048 to 0.032 and 0.030, representing reductions of 33.3 and 37.5%. These patterns support partial mediation through both the economic-burden channel and the primary-service supply channel. Figure 8 summarizes the mechanism. The reimbursement reform lowers OOPRatio and raises Supply; both channels then contribute to a higher probability of choosing lower-level care.

**Table 6:** Mechanism test for the effect on provider choice.

Variable	Model 1 (oopratio)	Model 2 (supply)	Model 3 (primarychoice+oopratio)	Model 4 (primarychoice+supply)
Treat × post	−0.045*** (0.005)	0.35*** (0.05)	0.032*** (0.006)	0.030*** (0.006)
OOPRatio	—	—	−0.18*** (0.02)	—
Supply	—	—	—	0.025*** (0.003)
Control variables (individual + disease + institution)	Yes	Yes	Yes	Yes
Individual and time fixed effects	Yes	Yes	Yes	Yes
Observations	420,000	420,000	420,000	420,000
R^2^	0.120	0.080	0.140	0.135

Compared with the baseline coefficient, the Treat x Post coefficients in Models 3 and 4 decline by 33.3 and 37.5%, respectively. This indicates that out-of-pocket burden and primary-service supply partially mediate the policy effect. *p < 0.10, **p < 0.05, and ***p < 0.01.

**Figure 8:**
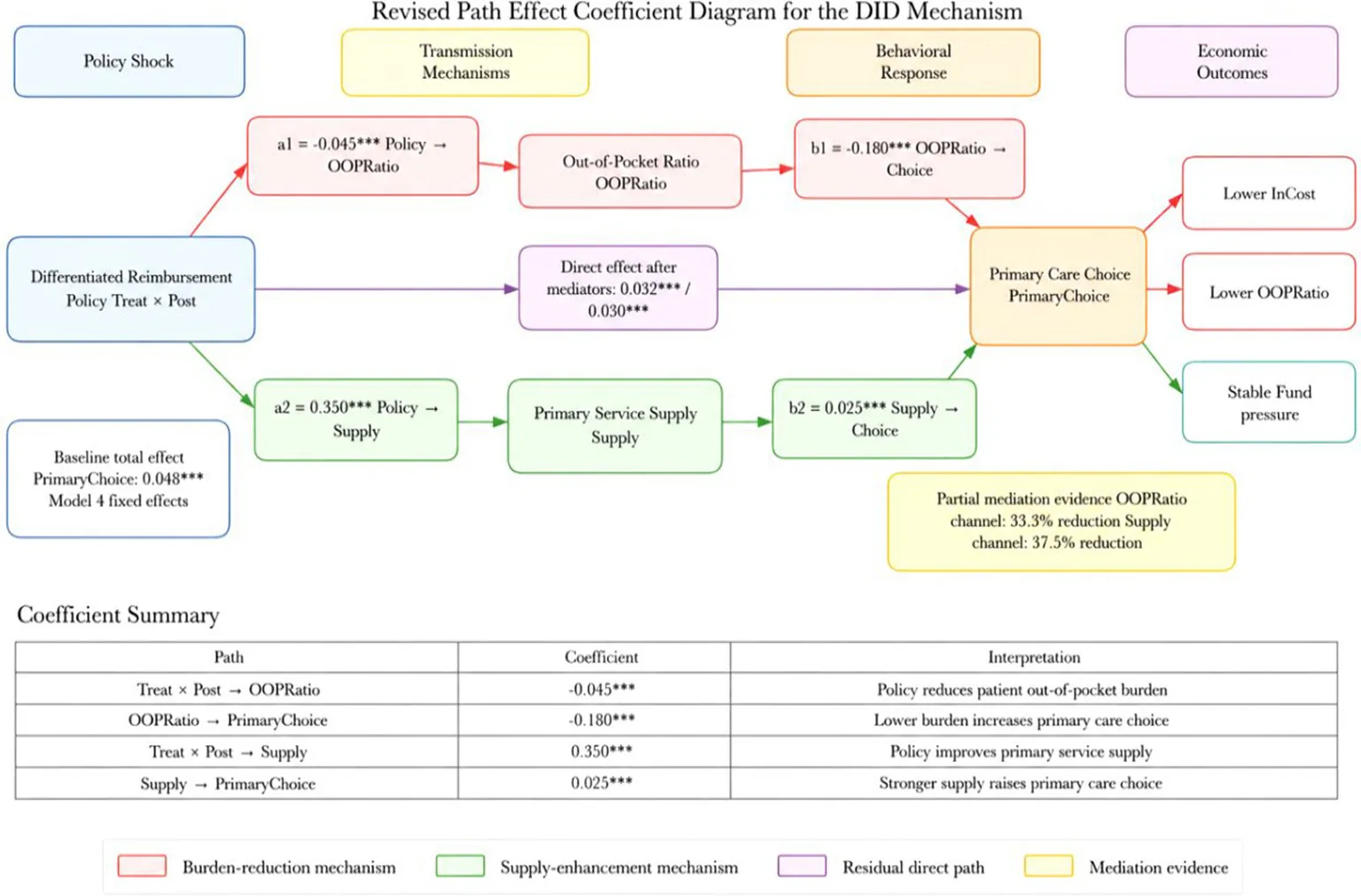
Path coefficients for the DID mechanism.

Table 7 reports heterogeneous policy responses across disease type, income level and urban–rural status. Chronic-disease patients show a stronger response because repeated management needs make them more sensitive to payment changes and more suitable for lower-level care. In contrast, severe cases rely more on specialist expertise, medical equipment and perceived treatment safety, which may reduce their response to reimbursement incentives. The stronger response among low-income patients reflects not only financial constraints but also greater sensitivity to out-of-pocket burden. The weaker response among rural patients may be associated with transportation barriers, limited primary-care accessibility and differences in healthcare information availability. Overall, heterogeneous effects are shaped by disease characteristics, socioeconomic vulnerability and local healthcare conditions.

**Table 7:** Heterogeneity results: Treat x Post coefficients and standard errors.

Grouping dimension	Subgroup	Lower-level provider choice	lnCost	OOPRatio	Observations
Disease type	Chronic-disease patients	0.065*** (0.010)	−0.10*** (0.015)	−0.06*** (0.008)	126,000
Disease type	Acute-disease patients	0.035** (0.014)	−0.06** (0.020)	−0.04** (0.010)	210,000
Disease type	Severe-case patients	0.012 (0.009)	−0.03 (0.025)	−0.02 (0.015)	84,000
Income group	Low-income group	0.070***(0.012)	−0.09*** (0.018)	−0.07*** (0.009)	140,000
Income group	Middle-income group	0.045*** (0.010)	−0.07***(0.015)	−0.05*** (0.007)	168,000
Income group	High-income group	0.020* (0.011)	−0.04* (0.020)	−0.03*(0.012)	112,000
Residence status	Urban patients	0.050*** (0.008)	−0.08*** (0.012)	−0.05*** (0.006)	252,000
Residence status	Rural patients	0.030** (0.013)	−0.06** (0.020)	−0.04** (0.010)	168,000

The table reports the coefficient of Treat x Post, with standard errors in parentheses. ***p < 0.01, **p < 0.05, *p < 0.1. Disease type, income level and urban–rural status are separate grouping dimensions; the groups within each dimension sum to the full sample.

Figure 9 displays the subgroup patterns. The increase in lower-level provider choice is larger for chronic-disease and low-income patients, while the cost and OOPRatio effects remain directionally consistent across groups.

**Figure 9:**
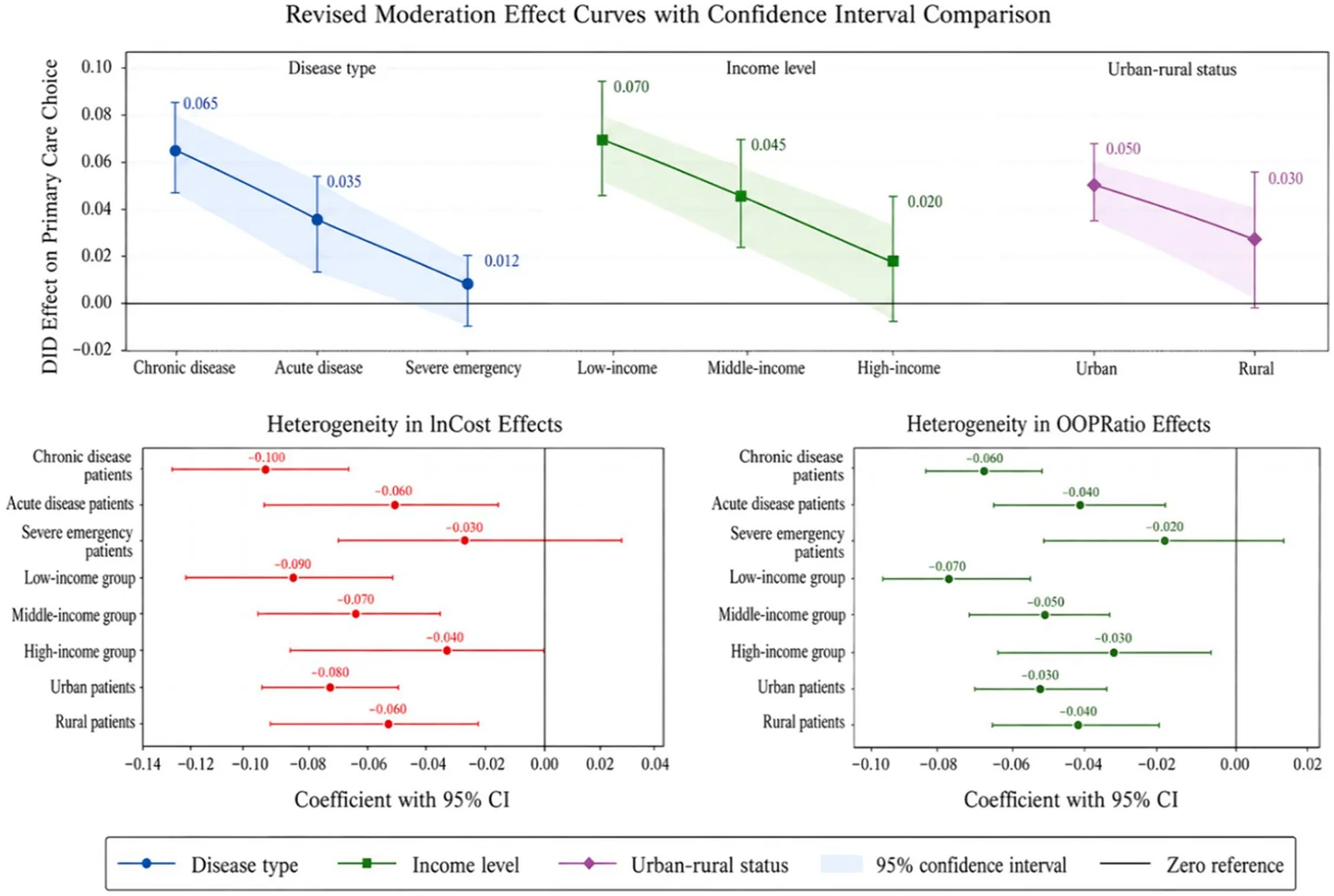
Heterogeneity curves and confidence intervals.

### 4.4 Robustness checks and summary of results

Table 8 evaluates the stability of the baseline results under several alternative specifications. The tests include redefining the treatment group, replacing the dependent variable, estimating marginal effects with logit and probit models, winsorizing the top and bottom 1% of expenditure variables, adding regional and institutional fixed effects, and conducting a placebo test. Across these checks, the PrimaryChoice coefficient remains positive and significant, while the lnCost and OOPRatio coefficients remain negative and significant. In the placebo test, the policy date is moved six months earlier, and the core coefficients become insignificant. This reduces the likelihood that the estimates are driven by pre-policy trends or unrelated contemporaneous shocks. The robustness evidence therefore supports the reliability of the main findings. In addition, the dynamic DID results show no significant differential trends between treatment and control groups before implementation, while placebo tests and alternative specifications further support the stability of the baseline estimates.

**Table 8:** Robustness check results.

Robustness test method	Lower-level provider choice	lnCost	OOPRatio	Observations
Benchmark model (LPM)	0.048*** (0.007)	−0.082*** (0.012)	−0.051*** (0.006)	420,000
Replace processing group definition	0.045***(0.008)	−0.078*** (0.013)	−0.048*** (0.007)	400,000
Replace the explained variable (base layer only)	0.038*** (0.006)	--	—	420,000
Logit model (marginal effect)	0.042*** (0.006)	--	--	420,000
Probit model (marginal effect)	0.043*** (0.006)	--	--	420,000
Eliminate the extreme value of 1% above and below the cost variable	0.047*** (0.007)	−0.079***(0.011)	−0.049*** (0.006)	411,600
Fixed effect of joining regions	0.046*** (0.007)	−0.080*** (0.012)	−0.050*** (0.006)	420,000
Fixed effect of joining medical institutions	0.044*** (0.008)	−0.076*** (0.013)	−0.047*** (0.007)	420,000
Placebo test (leave policy time)	0.006(0.007)	−0.010 (0.012)	−0.004 (0.006)	420,000

***p < 0.01, **p < 0.05, *p < 0.1. Standard errors are shown in parentheses. A dash indicates that the corresponding variable is not involved in the test. The placebo test sets a false policy date in July 2023, six months before the actual implementation, and the insignificant estimates support the robustness of the baseline results.

Figure 10 presents the robustness estimates with floating intervals. The PrimaryChoice coefficient stays positive under alternative specifications, whereas the placebo estimate is close to zero. Figure 11 reports residual diagnostics for the empirical specification. The Q-Q plot follows the normal reference line in the centre of the distribution, with only limited deviation in the tails.

**Figure 10:**
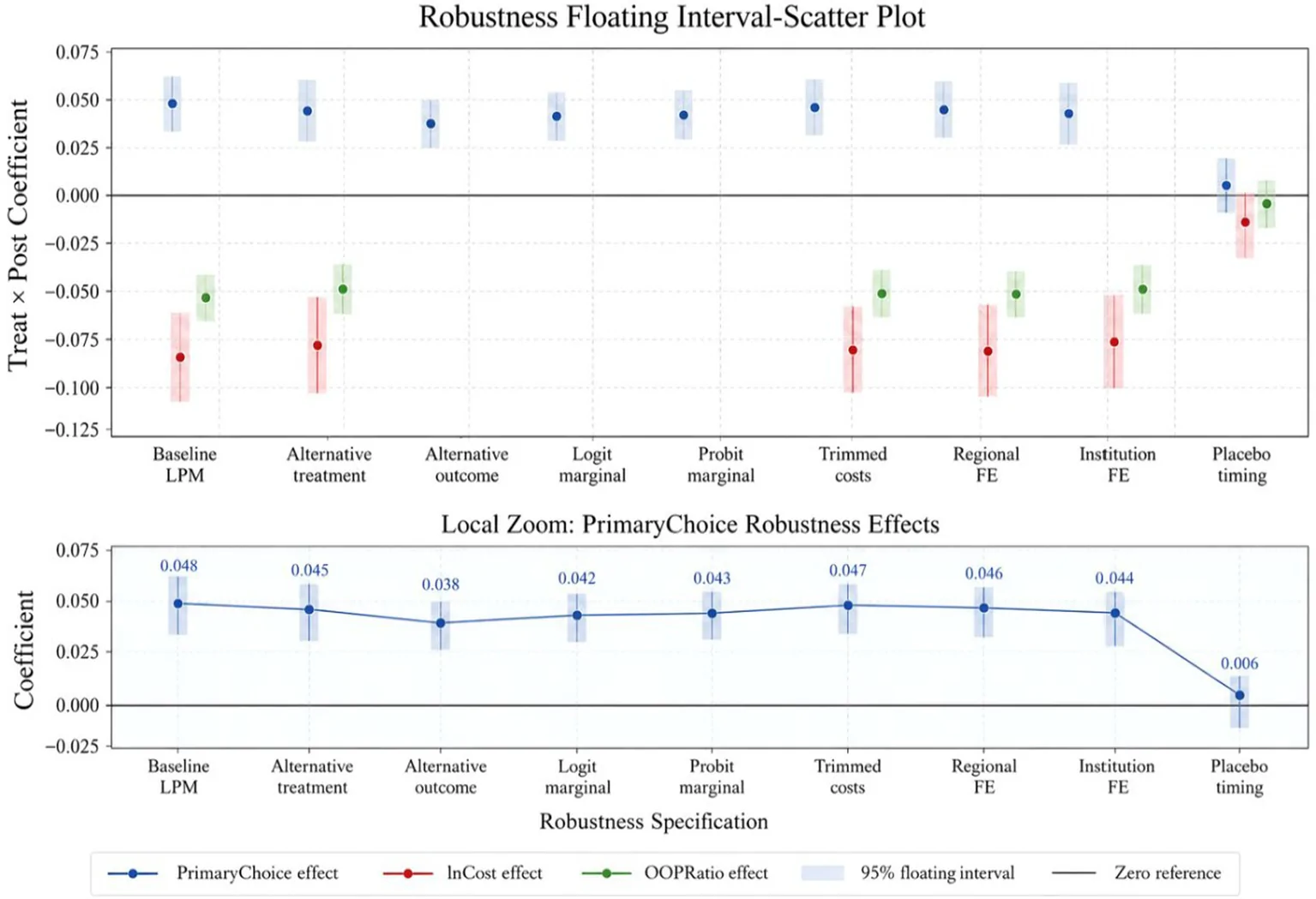
Robustness estimates with floating intervals.

**Figure 11:**
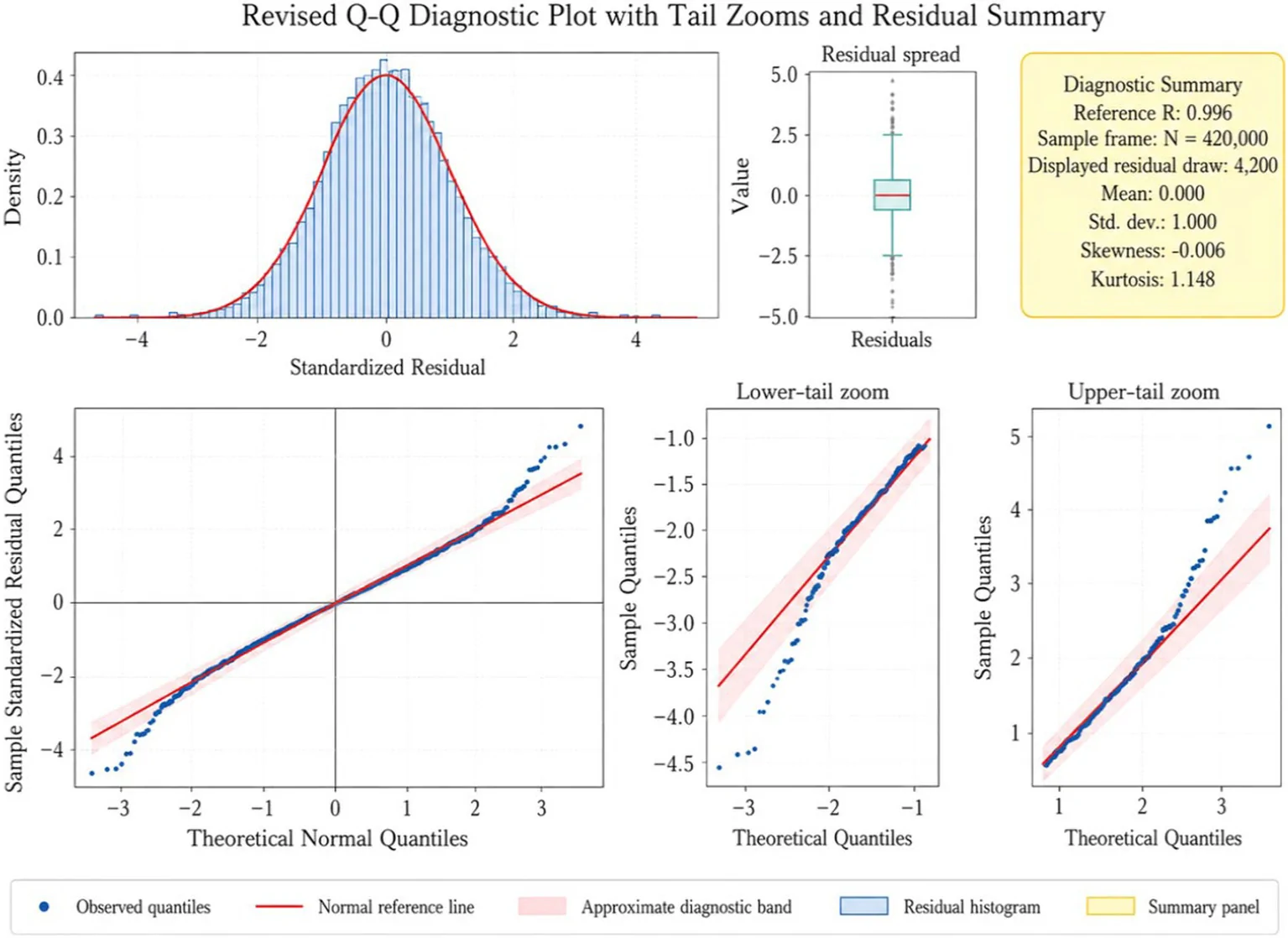
Q-Q diagnostic plot with tail details and residual summary.

Overall, the evidence shows that differentiated reimbursement significantly increases the use of primary or secondary institutions and reduces per-visit expenditure and patient payment burden. The mechanism analysis indicates that the effect is transmitted through lower out-of-pocket ratios and stronger primary-service supply. Heterogeneity analysis shows larger responses among chronic-disease patients, low-income groups and urban patients. Robustness checks confirm that these conclusions are not dependent on a single model, sample processing method or variable definition. The findings therefore support a more integrated reform strategy combining reimbursement incentives with primary-care capacity building and hierarchical diagnosis.

## 5 Discussion

### 5.1 Main findings and theoretical interpretation

Using provincial claims and institutional data from December 2022 to August 2024, this study identifies the effect of differentiated reimbursement on provider choice and medical expenditure. The baseline estimates show that the probability of choosing a primary or secondary institution rises by 4.8 percentage points after implementation. This indicates that reimbursement differences can redirect part of patient demand by changing relative prices. The cost model further shows a 7.9% reduction in per-visit expenditure and significant declines in both the level and share of individual payment, while insurance-fund payment does not increase significantly. In other words, the reform reduces the patient’s financial burden and improves the expenditure structure without producing clear additional pressure on the fund.

Table 9 connects the five hypotheses with the empirical evidence. H1, H2, H3 and H4 are supported by the baseline regression, heterogeneity analysis and mechanism test. H5 is only partially supported: the evidence confirms an overall reduction in costs, but the offsetting behavior of tertiary hospitals is discussed mainly from the perspective of provider response and would require more detailed item-level service data for direct verification.

**Table 9:** Verification of research hypotheses.

Hypothesis	Verification result	Interpretation
Differentiated reimbursement policy boosts choice of primary/lower-level institutions	Supported	Policy increases choice probability by ~4.8 pp.; reimbursement incentives guide patient diversion.
Guiding effect of policy is stronger for chronic vs. acute/critical patients	Supported	Chronic patients (coefficient: 0.065) are more responsive—their repeated care needs suit primary care.
Policy reduces single medical expenses and out-of-pocket burden	Supported	Reduces lnCost (−0.082), TotalCost (−150), OOPAmount (−120), OOPRatio (−0.051); no clear fund payment pressure expansion.
Primary care supply capacity mediates policy’s effect on patient choice	Supported	Policy improves Supply index by 0.35; adding Supply reduces core coefficient from 0.048 → 0.030—supply strengthens diversion effect.

The mechanism results help explain why the policy works. A lower out-of-pocket ratio increases the relative affordability of lower-level care, while stronger primary-service supply increases patients’ confidence that these providers can meet their needs. Once these two variables are introduced, the policy coefficient falls noticeably, suggesting that differentiated reimbursement does not operate through price alone. Instead, patient behavior changes when financial incentives and service capacity move in the same direction. This point is especially important for hierarchical diagnosis: a reimbursement advantage will have only limited attraction if primary capacity is weak, but it becomes more persuasive when local institutions can provide continuous and reliable services.

### 5.2 Relationship with existing studies

The findings are broadly consistent with recent work on medical-insurance payment reform and hierarchical diagnosis. Insurance incentives can alter patient flows, but their effect is conditioned by disease type, income and service capacity. The strongest response among chronic-disease patients confirms that reimbursement differentiation is most suitable for services with repeated visits, manageable clinical risk and a need for continuity. It should not be interpreted as a tool for limiting the reasonable use of high-level hospitals by patients with acute or severe conditions.

This study also adds evidence on the importance of supply-side conditions. The stronger response among low-income patients shows that reimbursement changes can have a redistributive effect by reducing financial barriers. The weaker response among rural patients suggests that distance, information access, doctor availability and equipment conditions may prevent patients from fully benefiting from the policy. Differentiated reimbursement should therefore be implemented together with primary-care capacity building, medical-consortium cooperation, referral reform and payment reform.

### 5.3 Policy implications

Firstly, reimbursement rules should be adjusted dynamically by disease type and patient need. For chronic diseases, common illnesses, rehabilitation and follow-up services that primary institutions can manage, the reimbursement advantage of lower-level care can be strengthened together with family-doctor contracts, long-term prescriptions and chronic-disease management. For acute, severe and complex conditions, policy should respect patients’ need for high-level hospitals and focus instead on early identification, rapid referral and coordination between institutions, rather than relying on reimbursement gaps to force diversion.

Secondly, insurance incentives need to be paired with stronger primary-care capacity. The mechanism results show that service supply is a key condition for the policy to take effect. Reform should therefore include doctor allocation, equipment upgrading, medicine availability, service-quality assessment and two-way referral efficiency, not only the reimbursement rate. In rural and resource-weak areas, medical alliances, telemedicine and support from higher-level hospitals should be used to strengthen local capacity and reduce urban–rural differences in policy benefits.

Thirdly, payment constraints and quality supervision should be strengthened for high-level hospitals. Patient diversion may reduce some tertiary-hospital volume, but high-level providers may respond by increasing tests, procedures or technical services. Health-insurance authorities should therefore combine differentiated reimbursement with DRG/DIP payment, total budget management, clinical pathways and performance assessment to limit supplier-induced offsetting behavior and protect the cost-control effect. Recent evidence on case-based payment reform and the removal of pharmaceutical co-payments for chronic conditions at the primary-care level further supports coordinating reimbursement incentives with provider-payment reform and primary-care strengthening (37, 38).

### 5.4 Limitations and future research

Several limitations should be noted. First, the sample comes from one province, so the conclusions should be tested with data from more regions before broader generalization. Second, the study period from December 2022 to August 2024 is suitable for identifying short- and medium-term effects, but it cannot fully capture long-term changes in patient habits, trust in primary institutions or strategic adjustment by hospitals. Third, some factors that may affect provider choice, such as family care capacity, travel distance, health preferences and physician reputation, cannot be fully observed in the available data. Future research could combine multi-provincial panels, spatial econometric methods and longer follow-up data to examine regional diffusion, persistence and equity effects.

## 6 Conclusion

This paper evaluates differentiated health-insurance reimbursement using provincial settlement records, institutional operating data and patient survey information from December 2022 to August 2024. The DID estimates show that the policy significantly increases the probability of choosing primary or secondary institutions and reduces per-visit expenditure, individual payment and the out-of-pocket ratio. Insurance-fund payment does not rise significantly, suggesting that the reform eases patient burden without creating evident additional fund pressure.

The effect is transmitted mainly through two mechanisms: a lower patient payment share, which makes lower-level care more financially attractive, and improved primary-service supply, which strengthens the ability of local institutions to receive patients. Heterogeneity analysis shows stronger responses among chronic-disease patients, low-income patients and urban patients, and weaker responses among acute or severe cases and rural patients. These results confirm that differentiated reimbursement has clear boundaries and depends on disease characteristics, economic constraints and service conditions.

Overall, differentiated reimbursement is an important instrument for promoting hierarchical diagnosis and improving the allocation of medical resources. Its effectiveness, however, depends on coordinated reforms in primary-care capacity, referral management, payment methods and quality supervision. Future policy should move beyond a single reimbursement-rate adjustment toward an integrated governance model that combines price incentives, service capacity, payment constraints and quality assurance.

## Data Availability

The original contributions presented in the study are included in the article/supplementary material, further inquiries can be directed to the corresponding author.
